# Integrated analysis of whole blood oxylipin and cytokine responses after bacterial, viral, and T cell stimulation reveals new immune networks

**DOI:** 10.1016/j.isci.2023.107422

**Published:** 2023-07-18

**Authors:** Etienne Villain, Aurélie Chanson, Malwina Mainka, Nadja Kampschulte, Pauline Le Faouder, Justine Bertrand-Michel, Marion Brandolini-Bulon, Bruno Charbit, Munyaradzi Musvosvi, Nicole Bilek, Thomas J. Scriba, Lluis Quintana-Murci, Nils Helge Schebb, Darragh Duffy, Cécile Gladine, the Milieu Interieur Consortium, Laurent Abel, Andres Alcover, Hugues Aschard, Philippe Bousso, Nollaig Bourke, Petter Brodin, Pierre Bruhns, Nadine Cerf-Bensussan, Ana Cumano, Christophe D’Enfert, Ludovic Deriano, Marie-Agnès Dillies, James Di Santo, Gérard Eberl, Jost Enninga, Jacques Fellay, Ivo Gomperts-Boneca, Milena Hasan, Gunilla Karlsson Hedestam, Serge Hercberg, Molly A. Ingersoll, Olivier Lantz, Rose Anne Kenny, Mickaël Ménager, Hugo Mouquet, Cliona O'Farrelly, Etienne Patin, Sandra Pellegrini, Antonio Rausell, Frédéric Rieux-Laucat, Lars Rogge, Magnus Fontes, Anavaj Sakuntabhai, Olivier Schwartz, Benno Schwikowski, Spencer Shorte, Frédéric Tangy, Antoine Toubert, Mathilde Touvier, Marie-Noëlle Ungeheuer, Christophe Zimmer, Matthew L. Albert, Darragh Duffy, Lluis Quintana-Murci

**Affiliations:** 1 Institut Pasteur, Université Paris Cité, Translational Immunology Unit, Paris, France; 2 Université Clermont Auvergne, INRAE, UNH, Clermont-Ferrand, France; 3 Chair of Food Chemistry, Faculty of Mathematics and Natural Sciences, University of Wuppertal, Wuppertal, Germany; 4 MetaToul, MetaboHUB, Inserm/UPS UMR 1048-I2MC, Institut des Maladies Métaboliques et Cardiovasculaires, 31400 Toulouse, France; 5 Université Clermont Auvergne, INRAE, UNH, Plateforme D’Exploration Du Métabolisme, MetaboHUB Clermont, Clermont-Ferrand, France; 6 Institut Pasteur, Université Paris Cité, CBUTechS, Paris, France; 7 South African Tuberculosis Vaccine Initiative (SATVI), Division of Immunology, Department of Pathology and Institute of Infectious Disease and Molecular Medicine, University of Cape Town, Cape Town, South Africa; 8 Institut Pasteur, Université Paris Cité, CNRS UMR2000, Human Evolutionary Genetics Unit, Paris, France; 9 Collège de France, 75005 Paris, France

**Keywords:** Immunology, Immune response, Microbiology

## Abstract

Oxylipins are major immunomodulating mediators, yet studies of inflammation focus mainly on cytokines. Here, using a standardized whole-blood stimulation system, we characterized the oxylipin-driven inflammatory responses to various stimuli and their relationships with cytokine responses. We performed a pilot study in 25 healthy individuals using 6 different stimuli: 2 bacterial stimuli (LPS and live BCG), 2 viral stimuli (vaccine-grade poly I:C and live H1N1 attenuated influenza), an enterotoxin superantigen and a Null control. All stimuli induced a strong production of oxylipins but most importantly, bacterial, viral, and T cell immune responses show distinct oxylipin signatures. Integration of the oxylipin and cytokine responses for each condition revealed new immune networks improving our understanding of inflammation regulation. Finally, the oxylipin responses and oxylipin-cytokine networks were compared in patients with active tuberculosis or with latent infection. This revealed different responses to BCG but not LPS stimulation highlighting new regulatory pathways for further investigations.

## Highlights

• Oxylipins are important immunomodulating mediators
• Bacterial, viral, and T cell immune responses show distinct oxylipin signatures
• Integrating oxylipin and cytokine responses reveals new immune networks
• Active tuberculosis or latent infection induce distinct oxylipin responses

## Graphical abstract

**Figure:**
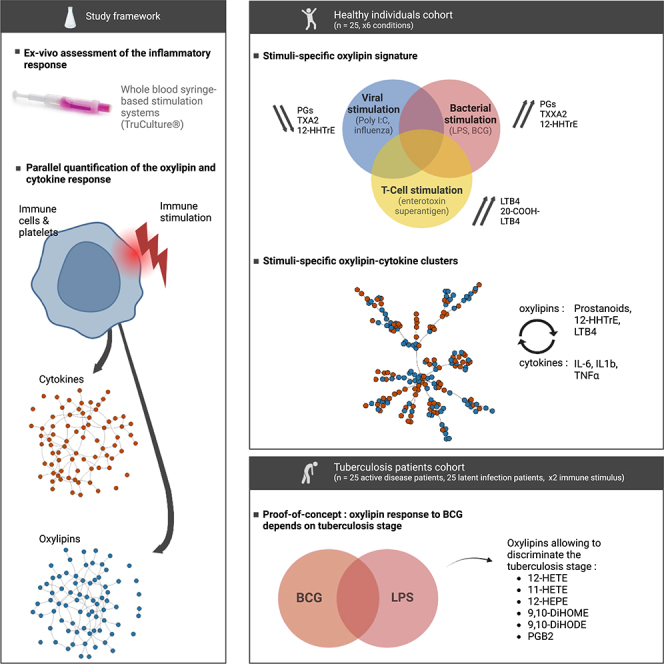

## Introduction

The inflammatory response is a protective immune response against infection or injury. This two-phase process starts with an acute response to eliminate the original insult, and is terminated through a resolutive phase to restore homeostasis. A timely succession of these two phases is crucial to ensure that the insult will be eliminated without causing excessive collateral damage. For that, the host produces a wide range of inflammatory mediators including cytokines, chemokines, and oxylipins (including eicosanoids). These later mediators are lipid metabolites produced from the oxygenation of polyunsaturated fatty acids (PUFAs) through a complex network of biochemical reactions involving over 50 unique and cell-specific enzymes.^1^ Briefly, after activation by Ca^2+^ dependent and independent mechanisms, cytosolic phospholipase A2 (cPLA2) releases the PUFAs from membrane phospholipids. Then, PUFAs are converted to a wide range of oxylipins via four major pathways: the cyclooxygenase (COX) pathway producing prostaglandins (PGs) and thromboxanes (Txs); the lipoxygenase (LOX) pathway producing hydroperoxy-PUFAs and leukotrienes (LTs); the cytochrome P450 pathway (CYP), primarily producing epoxy-PUFAs^2^ and the non-enzymatic pathway producing various hydro(pero)xy-PUFAs, epoxy-PUFAs^3^ as well as iso- and neuroprostanes. Importantly, some oxylipins require the interaction between different cell types to be produced through transcellular biosynthesis. For instance, this has been shown with platelets that interact with monocytes and neutrophils to produce thromboxanes (TXA_2_), leukotrienes (LTC_4_), and lipoxins (LXA_4_ and LXB_4_).^4^ For the last 40 years, extensive knowledge has accumulated on the role of oxylipins in the regulation of inflammation and infection.^5^ Notably, prostanoids and leukotrienes (i.e., PGE_2_, PGI_2,_ and LTB_4_ derived from arachidonic acid) have crucial roles in the acute inflammatory response as mediators of vasodilatation, oedema formation, vascular permeability, and PMN attraction.^6^^,^^7^

Induction of oxylipin synthesis has been shown to occur via the activation of toll-like receptors (TLR) by microbial- or pathogen-associated molecule patterns (MAMPs or PAMPS).^8^ Notably, the induction via TLR4 ligands such as LPS has been extensively studied showing a synergistic activation of both the cytosolic and soluble PLA2 leading to the induction of COX-2 and increased levels of PGE_2_.^9^^,^^10^ Although this is much less documented, other TLRs could be involved in oxylipin synthesis. This has been demonstrated in RAW264.7 macrophages stimulated with 16 different TLR agonists (inducing TLR 1, 2, 3, 4, 5, 6, 7, and 9). In this study, all TLRs induced oxylipin synthesis, especially the COX products PGD_2_ and its dehydration products PGJ_2_, 15d-PGJ_2_ and 15d-PGD_2_.^10^ It was also shown that G-protein coupled receptors (GPCR) agonists (UDP and PAF) and purinergic activation by millimolar levels of ATP can induce oxylipin synthesis in RAW264.7 macrophages.^10^

Circulating immune cells and platelets are particularly well equipped enzymatically to produce a large array of oxylipins involved in the regulation of the inflammatory response. Notably, enzymes of the LOX pathway involved in the production of leukotrienes (LT, e.g., LTB_4_) are highly expressed in monocytes and neutrophils as well as platelets.^11^ The enzymes of the COX pathway as well as the downstream enzymes that produce prostanoids (i.e., PG and TX) are highly expressed in monocytes, as well as in T cells and B cells.

Oxylipins and cytokines can co-regulate each other, thus providing efficient feedback regulation between these two types of response. A relevant example is the IL-1β triggered generation of prostaglandin E_2_ (PGE_2_), which represents the central mechanism of fever response.^12^ Although interactions between oxylipins, cytokines, and chemokines have been reported in various inflammatory contexts,^13^ most studies have been based so far on unicellular models (mainly using macrophage cell lines) and focused only on a couple of well-known oxylipins (e.g., PGE_2_). This provides a very limited view of the complexity of the interactions between oxylipins and cytokines during the inflammatory response and represents an important research gap for the understanding of inflammation and infection.^1^ To address this limitation we used a standardized whole-blood stimulation system maintaining total leukocytes and platelets in a plasma matrix (TruCulture)^14^ from which we comprehensively characterized the oxylipin responses after bacterial, viral, and T cell stimulation. The oxylipin responses were then integrated with the cytokine responses previously assessed from paired samples of healthy donors^15^ as well as from patients with latent or active tuberculosis.^16^ This approach integrating both the cellular and the molecular complexity of the inflammatory response provides a unique way to study immune networks in health and disease.

## Results

### Bacterial, viral, and T cell immune responses have distinct oxylipin signatures

**Figure 1:**
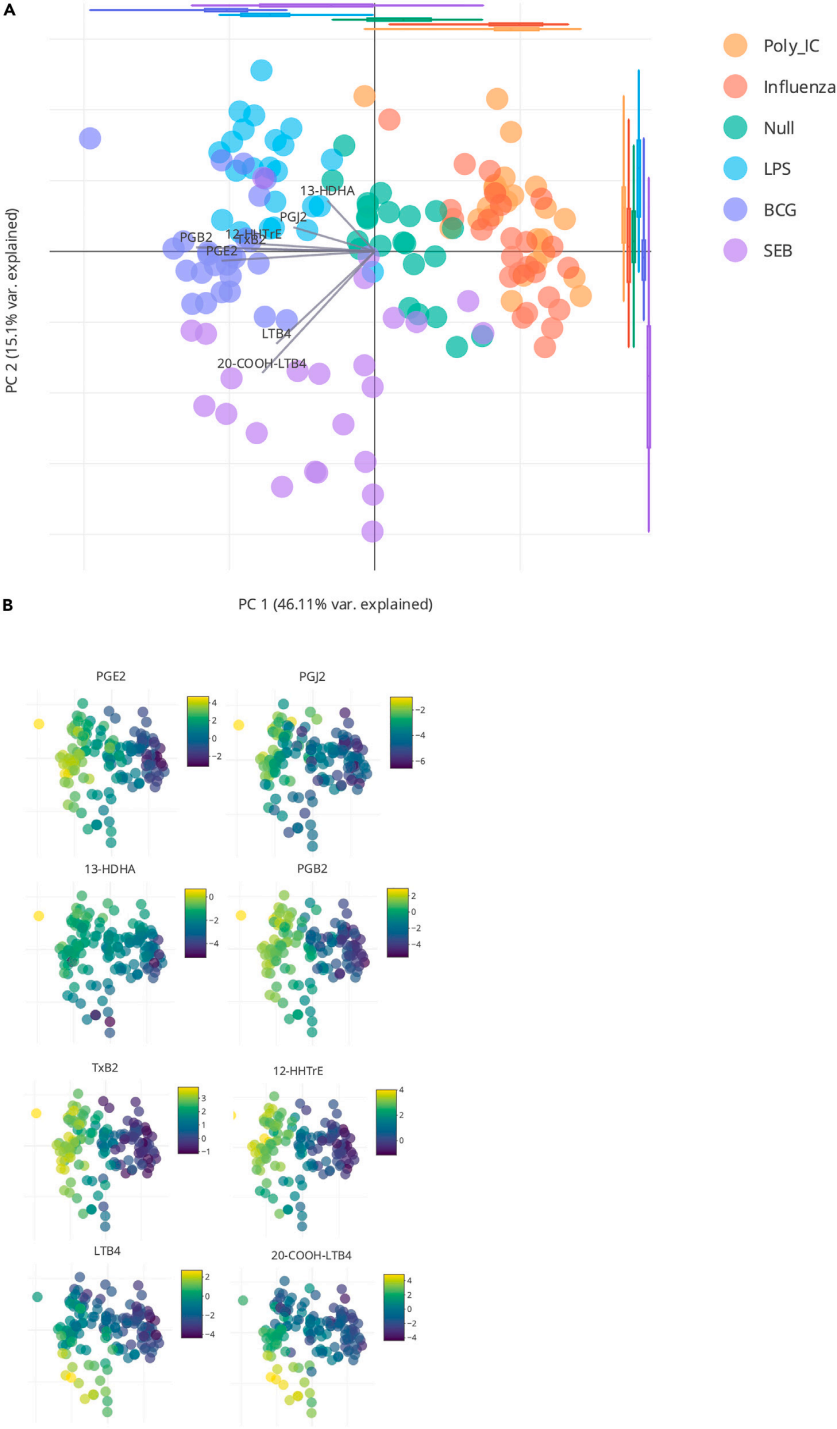
Oxylipin response after whole blood stimulation (A) PCA of oxylipins measured by MS LC-MS/MS at 22 h after whole blood stimulation with Poly:IC, Influenza virus, LPS, BCG, SEB, and Null condition as indicated by the color code. (B) Heatmap overlay on the PCA for the quantitative levels of PGE2, 13-HDHA, TxB_2_, LTB_4_, PGJ_2_, PGB_2_, 12-HHTrE, and 20-COOH-LTB_4_. n = 25.

Principal component analysis (PCA) of the oxylipin response of whole blood cells stimulated with bacterial (LPS and BCG), viral (poly I:C and Influenza) or superantigen enterotoxin (SEB) shows a distinct stimulus specific response (Figure 1A). The first two components (that explain, respectively, 46% and 15% of the total variance) show separation of the bacterial stimuli (on the left of the projection plan) from the viral ones (on the right) (Figure 1A). Both bacterial and viral stimulus clusters are distinct from the unstimulated control samples, located in the middle. A noticeable exception is the response to SEB superantigen stimulation that is separated from the other stimuli based on PC2. The distinction between the bacterial and viral stimuli by the first PCA component, is mainly driven by the induction of prostaglandins (PGB_2_, PGE_2_, and to a lesser extent PGJ_2_, PGE_1_, PGF_2_α) thromboxane B2 (TxB_2_), 12-HHTrE, leukotriene B_4_ (LTB_4_) and its derivative 20-COOH-LTB_4_ (Figures 1B and S1A). The distinction between the different bacterial stimuli, observable on the second component of the PCA, is mainly driven by leukotrienes (LTB_4_ and 20-COOH-LTB_4_) (Figure S1B). The SEB induced response is distinct from the other bacterial stimuli due to a stronger LTB_4_ response (Figure 1B), and to a lesser extent 12-HEPE, 11-HEPE and 10-HDHA, 14-HDHA, 11-HDHA, 13-HDHA (all omega-3 oxylipins) which are decreased in response to SEB. This likely reflects the strong T cell response induced by the SEB superantigen, which is not present following LPS stimulation and to a much weaker extent with BCG stimulation. Interestingly in the initial PCA the two viral stimuli, Influenza and poly I:C were indistinguishable, in contrast to previous results observed with induced cytokines^15^ or gene expression.^17^ Both of the viral responses were broadly characterized by reduced levels of specific oxylipins as compared to the Null control (Figures 1A and 1B).

**Figure 2:**
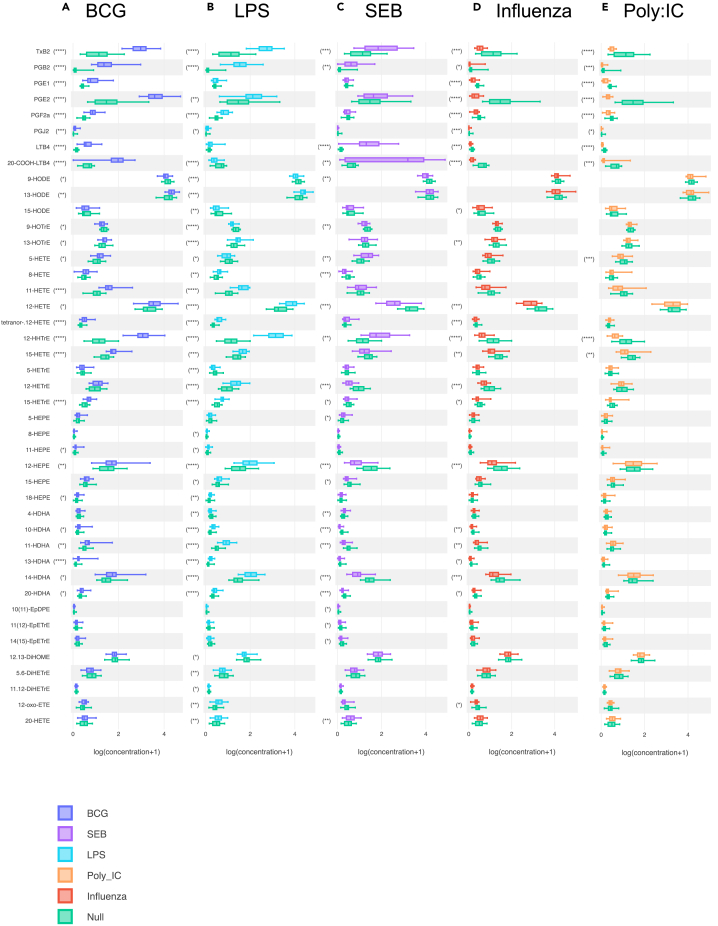
Stimulation specific oxylipin whole blood responses (A–E) Boxplots indicating the significantly induced oxylipin response for (A) BCG (B) LPS, (C) SEB, (D) Influenza, and (E) Poly I:C. in comparison with the Null condition. n = 25. Wilcoxon paired t-test vs. Null; ∗p < 0.05, ∗∗p < 0.01, ∗∗∗p < 0.001, ∗∗∗∗p < 0.0001.

The clusters formed by LPS and BCG separate well from the null and the viral stimulus (Figure 2), this distinction is mainly driven by the induction of prostanoids and many long chain hydroxy fatty acids (notably the DHA derived HDHA, visible on the Figure 2 first component composition, see Figure S1). Only LTB_4_ and 20-COOH-LTB_4_ have different signatures between LPS (low levels) and BCG (high levels), potentially reflecting the antigen specific T cell response induced by BCG. A second major cluster is determined by the SEB induced response, reflecting the results seen with the PCA (i.e., high levels of LTB_4_ and its metabolite 20-COOH-LTB_4_). The two viral stimuli (poly I:C and influenza) show very similar responses that were quantitatively opposite to the bacterial response (i.e., low levels of prostanoids and long chain hydroxy fatty acids).

Univariate analysis was used to precisely identify the most important shifts in the oxylipin signature after each stimulation. For each oxylipin, the stimulated condition was compared to the null with a Wilcoxon paired-t-test and an FDR correction applied (Figure 2, Table S3). This identified 43 oxylipins that were significantly different (FDR p < 0.05) to the Null in at least one stimulation condition. The most significant oxylipin changes were obtained with the bacterial stimuli (LPS and BCG) and were characterized by high levels of prostanoids. For instance, with LPS, the mean concentrations of TXB_2_, PGB_2_, PGF_2_α, and PGJ_2_ were, respectively, 6, 17, 2, and 2.5 times higher in comparison with the null samples. With BCG, the shift of prostanoids signature was even stronger with 8-fold, 17-fold, 3-fold, 8-fold, 2-fold, and 3-fold increases of TXB_2_, PGB_2_, PGE_1_, PGE_2_, PGF_2_α, and PGJ_2,_ respectively. Both bacterial stimuli were also characterized by a high level of 12-HHTrE (×10 and ×9 with LPS and BCG, respectively) and more modest shifts (∼2-fold increases in comparison with the null condition) of several hydroxy-fatty acids (e.g., 11-HETE, 11-, 13-, and 14-HDHA). BCG, but not LPS also induced increased levels of LTB_4_ and its metabolite 20-COOH-LTB_4_, while LPS but not BCG induced lower levels of the 5-LOX products (5-HETrE and 5-HETE). The viral stimuli (poly I:C and influenza) both induced very similar oxylipin signatures. However, these were diametrically opposed to the oxylipin signatures obtained with the bacterial stimuli. In comparison with the null condition, poly I:C and influenza samples were characterized by unchanged or lower levels of all quantified oxylipins in comparison with the null samples. In particular all prostanoids (TXB_2_, PGB_2_, PGE_1_, PGE_2_, PGF_2_α, PGJ_2_), LTB_4_ and its metabolite 20-COOH-LTB_4_, as well 12-HHTrE were reduced. Stimulation with superantigen SEB was associated with an intermediate oxylipin signature that was characterized by very high levels of LTB_4_ and 20-COOH-LTB_4_ (mean concentrations ×4,5 and ×33, respectively, in comparison with the null condition), high levels of TXB_2_ and PGB_2_ and 12-HHTrE (mean concentrations ×1,8 and ×2.4 and ×1.7, respectively, in comparison with the null condition) while several hydroxy-fatty acids (12-HETE, 12-HETrE, 12-HEPE, 10-HDHA, and 14-HDHA) exhibited low levels in comparison with the null samples.

### Bacterial, viral, and T cell immune responses involve multiple and consistent interactions between oxylipins and cytokines

**Figure 3:**
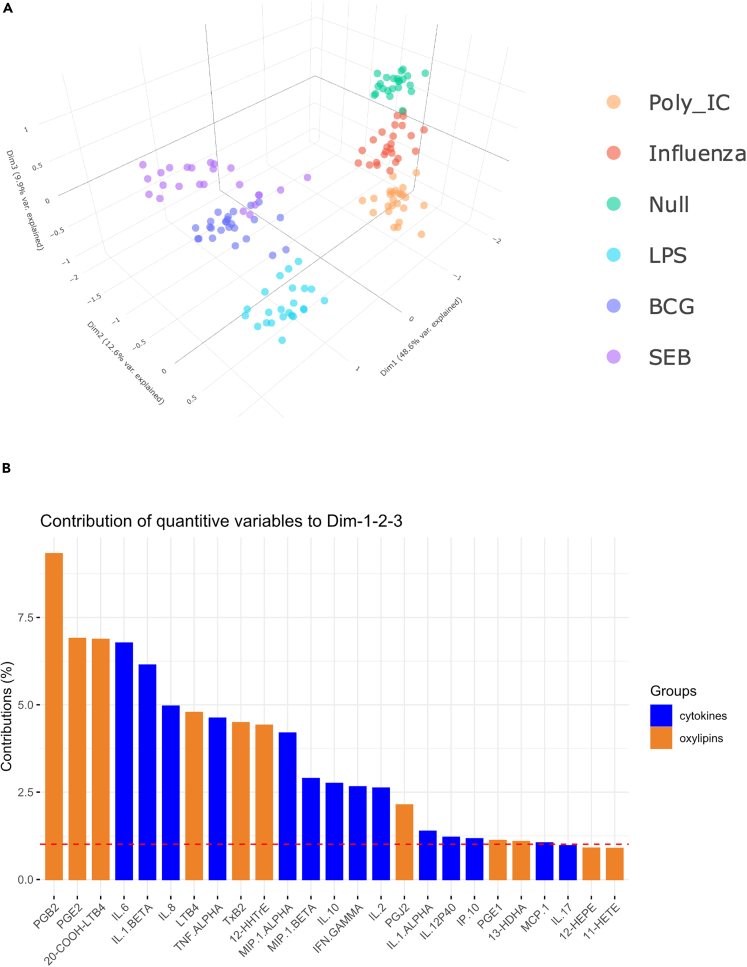
Integrative analysis of oxylipin and cytokine whole blood responses Multiple Factor Analysis (MFA) of cytokine and oxylipin concentrations measured at 22 h after whole blood stimulation with Poly:IC, Influenza virus, LPS, BCG, SEB, and Null condition, as indicated by the color code. (A) Scatterplot of individuals in the first three dimensions of the MFA (representing 71% of the explained variance). Individuals are colored according to type of stimulus, which has been added as an additional qualitative variable in the analysis. (B) Contribution of variables to the first three dimensions of the MFA. The red dotted line indicates the average value expected if the contributions of all variables were uniform. Cytokines are shown in blue color, and oxylipins in orange. n = 25.

The oxylipin dataset was combined with the cytokine dataset obtained from paired samples^15^ and analyzed using Multiple Factor Analysis (MFA). MFA is an extension of PCA tailored to handle different types of data obtained from the same observations (here oxylipins and cytokines).^18^ Figure 3A displays the scatterplot of individuals in the first three MFA components that represent, respectively, 49%, 13%, and 10% of the explained variance. It confirms that individuals are well separated according to the stimuli, with the six stimulation conditions forming six distinct clusters. Clustering according to the stimuli was confirmed by unsupervised HCA performed on the individuals’ MFA coordinates in the first three dimensions (data not shown). Furthermore, as previously observed in the PCA based on just the oxylipin responses (Figure 1), bacterial and viral clusters are well segregated. The addition of the cytokine dataset allows to enhance the separation between the two viral stimuli (poly I:C and influenza) and to further separate the SEB and null conditions. Of note, the distinction between the stimuli classes is primarily driven by oxylipins, notably PGB_2_, PGE_2,_ and 20-COOH-LTB_4_ that are the three variables that contribute the most to the first three dimensions of the MFA (Figure 3B).

**Figure 4:**
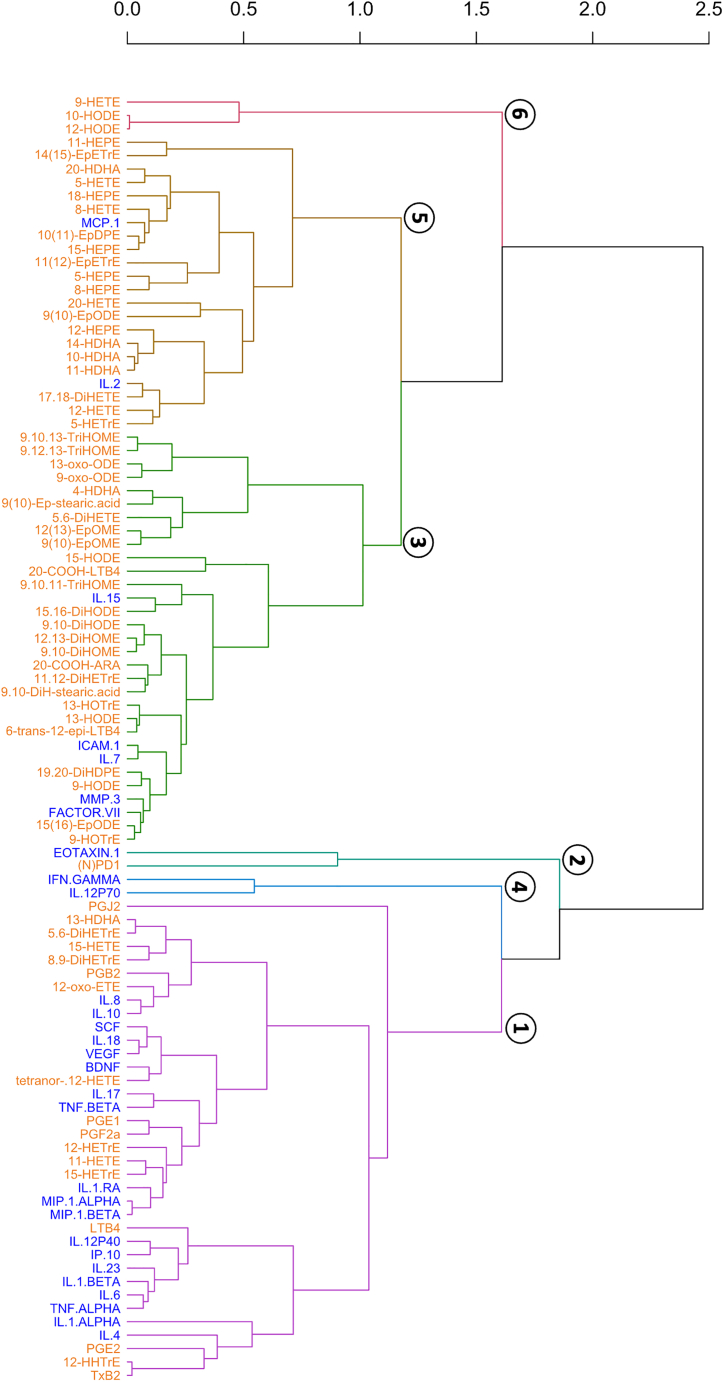
Integrative analysis of oxylipin and cytokine LPS whole blood response Dendrogram representing the clustering of the classes obtained in the hierarchical agglomerative cluster analysis performed on the coordinates of the variables (cytokines and oxylipins) in the first four dimensions of the Multiple Factor Analysis (MFA) applied on the sub-data set corresponding to the LPS stimulus. The first four dimensions of the MFA represent 57% of the explained variance. The dendrogram was obtained by using the euclidean distance as metric and by applying Ward’s method for class clustering. n = 25.

To further investigate the relationships within the oxylipin network, and between the oxylipin and cytokine networks, we performed MFA using the combined oxylipin and cytokine dataset from each stimulation group separately (i.e., LPS, BCG, influenza, poly I:C, and superantigen SEB). The MFA variable coordinates were then used to run HCA aiming at clustering oxylipins and cytokines. Molecules that were found in the same clusters were considered to display common signature patterns within the stimulation group of interest. Figure 4 represents the dendrogram obtained from the HCA performed with the LPS dataset and for which a six-class partition was retained. Dendrograms obtained with the other stimuli are shown in supplemental (Figure S2).

Cluster #1 of the LPS dendrogram (Figure 4) comprises 36 molecules including 17 oxylipins and 19 cytokines. More precisely, all prostanoids (i.e., PGJ_2_, PGB_2_, PGE_1_, PGF_2_α, PGE_2,_ and TXB_2_) were found in LPS-cluster #1 that also contained LTB_4_ and several long-chain hydroxy fatty acids (i.e., 13-HDHA, 15-HETE, 12-HETrE, 11-HETE, 15-HETrE). The cytokines found in LPS-cluster #1 comprise several interleukins (i.e., IL-8, IL-10, IL-18, IL-17, IL-1-RA, IL-12P40, IL-23, IL-1b, IL-1a, IL-6, and IL-4), TNFα and β, MIP-1α and β as well as BDNF, VEGF, and SCF. LPS-cluster #2 includes only two molecules, namely Eotaxin-1 and NPD1. Cluster #3 is composed of 31 molecules mostly represented by oxylipins (n = 26) with only 5 cytokines (IL-15, ICAM-1, IL-7, MMP-3, and Factor VII). Among the 26 oxylipins from LPS-cluster #3, we found a majority of short-chain species derived from 18-carbon fatty acids (i.e., 9,10,13-, 9,10,11- and 9, 12, 13-TriHOME, 13- and 9-oxo-ODE, 9(10)-Ep-stearic acid and 9,10-DiH-stearic acid, 9(10)- and 12(13)-EpOME, 15-HODE, 13-HOTrE, 9- and 13-HODE, 9-HOTrE, 9,10- and 15, 16-DiHODE, 1,10- and 12, 13-DiHOME and 15(16)-EpODE). LPS-cluster #4 only contains IFN-γ and IL-12p70, a tight cytokine network which we recently described in this whole blood stimulation system.^19^ Cluster #5 comprises 22 molecules including only two cytokines (MCP-1 and IL-2). Half of the oxylipins from this cluster are derived from long-chain omega-3 fatty acids (i.e., 5-, 8-, 11-, 12-, 15-, 18-HEPE, 10-, 11-, 14-, 20-HDHA and 10(11)-EpDPE). Finally, cluster #6 includes 9-HETE, 10- and 12-HODE.

**Figure 5:**
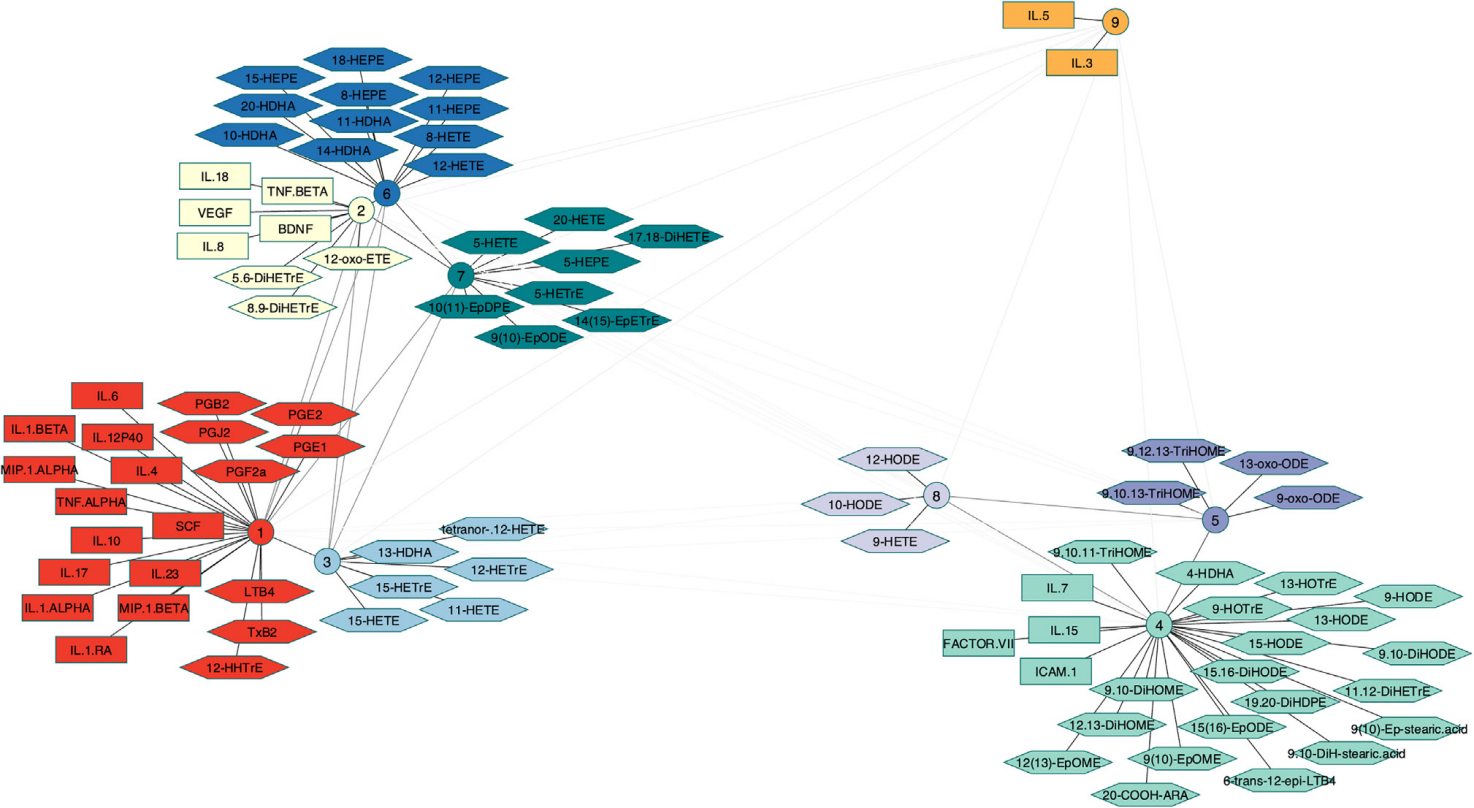
A proximity network for the oxylipin cytokine response Graphical representation of the network of proximities between molecules belonging to the nine clusters identified as similar between the LPS and BCG partitions. Distances between clusters are proportional to the inter-cluster dendrogram distances calculated from the dendrogram shown in Figure S3 (the darker the line, the smaller the dendrogram distance). Cytokines are shown as rectangles and oxylipins as hexagons. All molecules related to a cluster are equidistant to that cluster but have been placed differently to improve visualization. n = 25.

To assess the consistency of the oxylipin and cytokine clustering in response to bacterial stimuli, the clusters obtained with LPS and BCG were compared (see Table S4) by Multiple Correspondence Analysis (MCA) followed by HCA on the MCA response coordinates (Figure S3). The dendrogram distances between clusters of oxylipins/cytokines were then calculated and used to build a network of proximities between the clusters to facilitate visualization (Figure 5).^20^ Nine clusters (involving 87 oxylipins and cytokines) were identified as being similar between LPS and BCG supporting the consistency of these oxylipin and cytokine responses to bacterial stimuli. Clusters #1, 2, and 4 include both oxylipins and cytokines while clusters #3, 5, 6, 7, and 8 include only oxylipins, and cluster #9 only two cytokines (IL-5 and IL-3). Of note, cluster #9 was far from the other clusters (especially cluster #1) suggesting that, in response to the bacterial stimuli, IL-5 and IL-3 responded very differently than the other oxylipins and cytokines. Cluster #1 gathers all prostanoids, LTB_4_ and 12-HHTrE with several cytokines including IL-4, IL-6, IL-1α and β, TNF-α, IL-10, MIP-1α, MIP-1β that are characteristic of responses to BCG and LPS.^15^ The clusters gathering only oxylipins are consistent with their metabolic origins. For instance, cluster #7 gathers all 5-LOX metabolites (i.e., 5-HETE, 5-HEPE, and 5-HETrE) and clusters #4, 5, and 8 (that are relatively close to each other) include almost only octadecanoids (i.e., 18-carbons PUFAs derived oxylipins). Cluster #4 also includes IL-7, IL-15, ICAM-1, and the coagulation factor VII.

The consistency of the oxylipin and cytokine clustering in response to all stimuli was also assessed using a similar approach (Figure S4). Fifteen consistent clusters (involving 64 oxylipins and cytokines) were identified meaning that the molecules found in these clusters display common signature patterns whatever the immune stimulation. The all-stimuli network was relatively similar to the bacterial network (Figure 5) with the exception of prostaglandins that were not found in any of the fifteen consistent clusters. This is consistent with previous observations that prostaglandins are specifically induced following bacterial stimulation.

### Patients with active tuberculosis have differential oxylipin response to BCG, but not LPS stimulation

**Figure 6:**
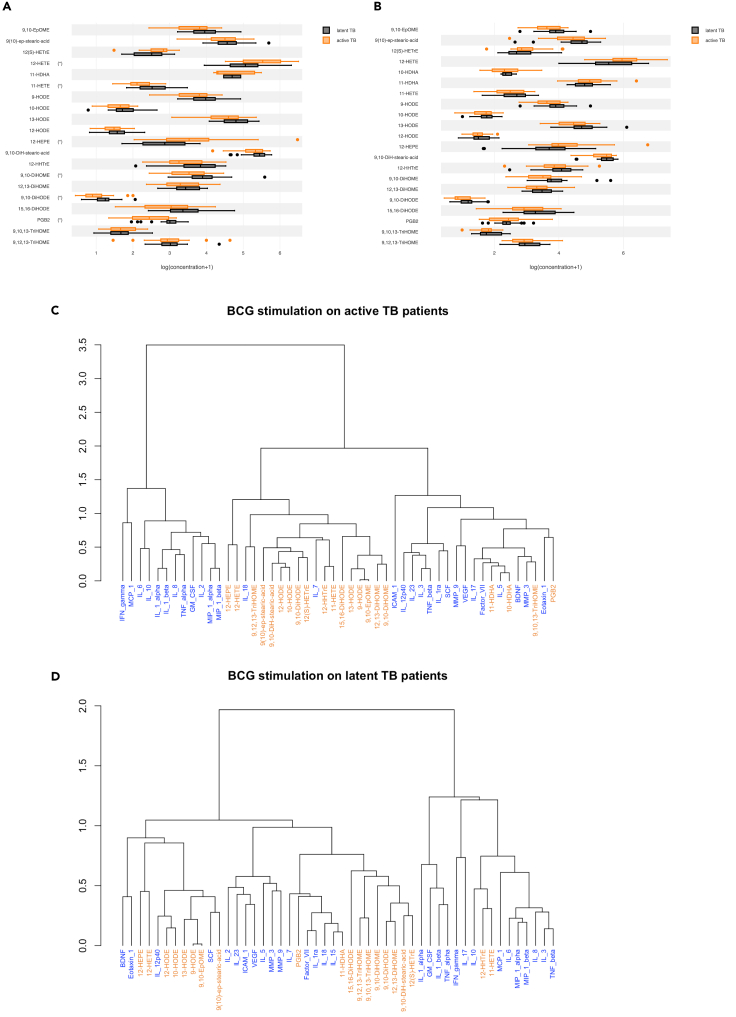
The oxylipin response in active TB infection (A–D) Boxplots indicating the significantly induced oxylipin response for (A) BCG (B) LPS between active TB patients and LTBI controls. Dendrograms representing the clustering of the classes obtained in the hierarchical agglomerative cluster analysis performed on the coordinates of the variables (cytokines and oxylipins) in the first four dimensions of the Multiple Factor Analysis (MFA) applied on the sub-data set corresponding to the BCG stimulus for (C) active TB and (D) LTBI controls. Stars indicate significant differences between the two groups as determined by a Wilcoxon test, FDR p < 0.05. n = 25 in each group.

To demonstrate the relevance of this approach in a disease setting, we took advantage of a previous TruCulture study of Tuberculosis (TB) patients, as the oxylipin response has been highlighted to be important in the host response to infection with *Mycobacterium. tuberculosis*.^21^ We compared patients with active Tuberculosis disease (TB) diagnosed by a PCR positive sputum test, and donors with latent infection (LTBI) as defined by a positive QFT test, recruited at SATVI, South Africa.^16^ We quantified oxylipins in the supernatants of whole blood stimulated with BCG and LPS. Of the 21 oxylipins quantified (i.e., >LLOQ), 6 were significantly different (FDR p < 0.05) between active TB and LTBI groups after BCG stimulation, but not after LPS stimulation. Specifically, the 12-LOX products (i.e., 12-HETE and 12-HEPE) were higher, and the PGE_2_ dehydratation product (i.e., PGB_2_) and to a lesser extent 11-HETE, 9,10-DiHOME, 9,10-DiHODE were lower in active TB patients compared to LTBI (Figure 6A). After LPS stimulation these oxylipins showed the same pattern as in the BCG condition, although none of the differences were statistically significant highlighting the disease relevancy of specific stimuli such as BCG in TB patients (Figure 6B).

To integrate this dataset with secreted cytokines, we performed MFA as previously described. 12-HEPE and 12-HETE clustered together in both groups, though in active TB patients they were associated with IL-18 (Figure 6C), an IL-1 family inflammatory cytokine previously shown to be elevated in TB,^22^ and IL-7. In contrast, in the LTBI group, 12-HEPE and 12-HETE were in a cluster with IL-12p40, BDNF, Eotaxin-1, and SCF (Figure 6D). PGB_2_ which was lower in active TB clustered with Eotaxin-1, in contrast to LTBI where it clustered with Factor VII, IL-7, IL-1RA, IL-18, and IL-15.

## Discussion

A better understanding of the inflammatory response is needed to improve the prevention and management of dysregulated inflammation. This is a central component in a wide range of chronic inflammatory diseases such as arthritis, atherosclerosis, as well as acute conditions such as sepsis and COVID-19. So far, most studies have focused on cytokines and chemokines that control key processes of host defense and inflammation. However, the cytokine network is only one component of the inflammatory response that is also regulated by an oxylipin network involving multiple cell types and hundreds of molecules. Interaction within and between both networks is essential to ensure an efficient and safe inflammatory response.^5^ In this study, we aimed to capture this complexity by implementing a comprehensive systems biology approach taking into consideration the cellular and molecular complexity of the oxylipin network and integrating it with the cytokine/chemokine network. Using this approach, we characterized the oxylipin secretome of whole blood cells in response to bacterial, viral, and T cell stimuli and established the first comprehensive oxylipin-cytokine network in the context of a healthy inflammatory response.

By using diverse stimuli in our experimental approach, we observed very distinct oxylipin signatures between the bacterial, viral, and SEB stimulation systems. Bacterial stimulation (LPS and BCG) induced high levels of prostanoids (i.e., COX oxylipins), most notably TXB_2_, that is primarily produced in platelets but also in activated monocytes/macrophages and dendritic cells (DCs).^23^ Other induced prostanoids in the bacterial responses include PGE_2_^24^ (and its dehydration product PGB_2_) and PGF_2_α which are ubiquitously produced, the dehydration product of PGD_2_ (i.e., PGJ_2_) that is mainly produced by mast cells as well as activated Th2 cells,^25^ and 12-HHTrE, a breakdown product of the COX primary product PGH_2_. The enhanced production of these oxylipins following LPS and BCG induction suggests a strong and persistent activation of the COX-2 and downstream prostanoid pathways. This is consistent with the known involvement of TLR4 signaling in the induction of PLA2, COX-2, and prostanoid synthases^26^ and the release of prostanoids.^27^ This is also consistent with our previous study using a similar whole blood stimulation system that showed pronounced production of TXB_2_, PGE_2_/PGB_2_, and PGF_2_α production after 24 h of LPS stimulation.^28^ Although BCG and LPS both signal via TLR4, slight differences of 5-LOX products were observed. In particular, BCG responses showed enhanced LTB_4_ production while LPS was associated with low levels of the 5-LOX products 5-HETrE and 5-HETE. This could be due to NO-mediated suppression of both 5-LO and FLAP function by prolonged exposure to LPS as previously reported in alveolar macrophages and in peripheral blood monocytes.^29^ This could also be due to the fact that BCG can signal through TLR2^30^ which has been shown to enhance LT released by human monocytes.^31^ BCG may also stimulate a low-level antigen specific T cell response, as all of the donors were vaccinated with BCG at birth. This is reflected by the overlap with the SEB response, most notably the lower induction of LTB_4_ metabolites. Viral stimulation (poly I:C and influenza virus) generated opposite oxylipin signatures with remarkably low levels of prostanoids and LT in comparison with the other stimulation conditions including the unstimulated samples. Similar segregation between LPS and poly I:C was reported for several cytokine/chemokine responses (e.g., IL-10, TNFα, IL-6) previously assessed on paired samples.^15^ The reduced level of COX-oxylipins are intriguing if we consider a previous study showing the activation of PLA2 and COX pathways in RAW264.7 macrophages incubated with poly I:C (25 μg/mL poly I:C for 18 h).^32^ However, our results are in line with a study of mice infected with various influenza virus strains showing systematic down-regulation of oxylipin synthesis after 24 h in the lungs of infected mice.^33^ The reduced levels of oxylipins following viral stimulation may also reflect kinetic differences in the whole blood response to these different stimulations, which will require further studies. Although influenza virus can activate TLR7/8 in addition to TLR3 and MDA5, no differences in oxylipin responses were observed between both viral stimulation conditions. However, it has been shown that TLR7/8 potentiates rather than activates oxylipin synthesis, notably on subsequent stimulation by fMLP, platelet-activating factor, and the ionophore A23187^34^ suggesting that in our influenza stimulation model, only TLR3/MDA5 has been engaged in the oxylipin response. After SEB stimulation, the oxylipin signature was distinguishable from the other responses mainly by a very strong induction of the LTB_4_ pathway. This specificity could be due to the TLR2-dependent LT production^31^ but also to T cell activation and the associated cytokine/chemokine response.^15^

Interestingly, when looking at the proximities of the oxylipin responses to the various stimuli, metabolic consistency was observed. For instance, in response to bacterial stimuli, all prostanoids from AA (i.e., PGB_2_, PGJ_2_, PGE_2_, TXB_2_) or all 5-LOX hydroxy fatty acids (i.e., 5-HETE, 5-HEPE, 5-HETrE) were clustered together. This suggests that, to understand and assess the inflammatory response and its dysregulation, it should be kept in mind that induction or inhibition of one oxylipin pathway can influence all metabolic relatives.

Multiple studies showed that oxylipins and cytokines can co-regulate each other. Our integrative multi-block analysis of combined oxylipin and cytokine data highlighted strong proximities between both responses. This identified known, as well as new potential interactions, within and between these two major families of inflammatory mediators. For instance, the proximity network built from both the LPS and BCG datasets identified one particularly interesting common cluster (cluster #1) gathering all prostanoids (PGE_2_/B_2_, PGJ_2_, PGE_1_, PGF_2_α, TXB_2_), LTB_4_ and 12-HHTrE and several cytokines including IL-4/6/8/10/17/23, IL-1β, IL12p40, TNF-α. The oxylipins and cytokines identified in this cluster were strongly induced by the bacterial stimuli supporting their proximity in the network.^15^ On the contrary, a small cluster including IL-5 and IL-3 was very distant from cluster #1, consistent with the low levels of these two cytokines after bacterial stimulation.^15^ Concomitant high levels of IL-1β and PGE_2_ is consistent with the well-described IL-1β-triggered generation of PGE_2_ that is a crucial mechanism of fever response and of macrophage antimicrobial activity during *M. tuberculosis* infection.^35^ This cross-talk between IL-1β and PGE_2_ has been reported in primary blood monocytes and relies on increased COX-2 gene expression.^36^^,^^37^ IL-1β was also described as a potent activator of LTB_4_ release in macrophages thus inducing neutrophil chemotaxis^38^ and of TXA_2_ by human PBMCs, while PGE_2_ also induces IL-8,^39^ IL12p40,^40^ and IL-17.^41^ The last discriminant oxylipin of cluster #1, 12-HHTrE, is a competitive product of TXA_2_ mainly produced in platelets and a natural ligand for leukotriene B_4_ (LTB_4_) receptor-2 (BLT2)^42^ but its roles in inflammatory responses remain uncertain. TNFα is another cytokine of the acute inflammatory response exerting some of its pro-inflammatory actions by inducing LTB_4_ release by neutrophils^43^ while LTB_4_ was shown to induce the production of TNFα in human monocytes. Enhancement of COX-2 gene expression and TXA_2_ production was also reported in primed human macrophages and human PBMCs exposed to TNFα.^44^ Concerning IL-6, a multifunctional cytokine involved in acute phase responses, there is no clear evidence that it may regulate oxylipin synthesis but, on the other hand, LTB_4_ has been shown to induce significant increased production of IL-6 in human monocytes.^45^ Intriguingly, high levels of COX- and LOX-oxylipins were also associated with high levels of IL-4 and IL-10, two anti-inflammatory cytokines that have been shown to suppress LPS-induced COX-2 and PGE_2_ production in neutrophils while inhibiting 5-LOX and COX-2 expression in DCs.^46^^,^^47^ However, PGE_2_ also induces IL10 production in DCs^48^ that may represent an additional cross-talk supporting the clusterizating of PGE_2_ and IL-10 in our datasets. Finally, the distance between cluster #1 and cluster #9 (IL-3 and IL-5) is somewhat inconsistent when considering that both cytokines are capable to induce FLAP and 5-LOX of murine mast cells^49^ and eosinophils^50^ but the differences of experimental procedure make these results incomparable with ours.

Our pilot study in TB patients and matched LTBI controls showed altered oxylipin responses and highlighted how disease relevant stimuli (e.g., BCG) could reveal interesting oxylipin-cytokine associated differences. The most striking observation was that, after stimulation with BCG, TB patients showed increased production of 12-LOX products (i.e., 12-HETE and 12-HEPE) concomitantly with a decreased production of PGE_2_ (assessed via its dehydration product PGB_2_) in comparison with the LTBI controls. Consistently, 12-HETE has recently been reported to be elevated in the plasma of TB patients, and specifically implicated in the recruitment of neutrophils which are considered major drivers of the immunopathology.^51^ Furthermore, SNPs in the myeloid/platelet-expressed 12-LOX (ALOX12) gene, which produces 12-HETE in humans were associated with higher risk of TB.^51^ Concerning PGE_2_, this is a key antimicrobial mediator in TB infection whose default of action has been associated with a higher risk of TB.^52^^,^^53^ Our integrative analysis also revealed new potential interactions between the oxylipin and cytokine responses in TB patients, notably between the 12-LOX products and IL-18 as well as between PGB_2_ and Eotaxin-1. In patients with active TB, IL-18 has been reported to be elevated in the plasma^54^ while in mouse models it was reported to be important for protection against infection.^55^

### Limitations of the study

This study had a few limitations. It was not possible to study a kinetic response of oxylipin production using different time points of *ex vivo* stimulation. This would be interesting to examine if viral stimuli induce different kinetics of oxylipin production as compared to bacterial and T cell stimuli. Although significant shifts of oxylipin responses could be observed in both studies, the relatively small sizes of our studies is a limitation and additional and larger studies should be performed to validate our findings and identify determinants of variable oxylipin responses.

### Conclusion

Oxylipins constitute a major bioactive lipid network to control immune responses to infection. Here, we have established for the first time the comprehensive oxylipin signatures of stimulated whole blood in the context of bacterial, viral, and T cell immunity in healthy individuals. This revealed that oxylipins are massively produced during these different immune responses and that the oxylipin signatures are specific to the type of immune response induced. Moreover, integrating the oxylipin responses with the cytokine responses provided unique interaction networks showing that interactions between oxylipins and cytokines are multiple and very consistent. Applying this approach in a pilot study of TB patients and matched LTBI controls highlighted specific alterations of the oxylipin response and oxylipin/cytokine network in TB patients that may identify new regulatory pathways for further investigation. Altogether, this work provides new knowledge on what is a healthy oxylipin response, its immune specificities and its integration with the cytokine response that could have a great clinical potential for the diagnosis and management of inflammation and infection.

## Consortia

The Milieu Intérieur Consortium (unless otherwise indicated, partners are located at Institut Pasteur, Paris) is composed of the following team leaders: Laurent Abel (Hôpital Necker), Andres Alcover, Hugues Aschard, Philippe Bousso, Nollaig Bourke (Trinity College Dublin), Petter Brodin (Karolinska Institutet), Pierre Bruhns, Nadine Cerf-Bensussan (INSERM UMR 1163—Institut Imagine), Ana Cumano, Christophe D’Enfert, Ludovic Deriano, Marie-Agnès Dillies, James Di Santo, Gérard Eberl, Jost Enninga, Jacques Fellay (EPFL, Lausanne), Ivo Gomperts-Boneca, Milena Hasan, Gunilla Karlsson Hedestam (Karolinska Institutet), Serge Hercberg (Université Paris 13), Molly A Ingersoll, Olivier Lantz (Institut Curie), Rose Anne Kenny (Trinity College Dublin), Mickaël Ménager (INSERM UMR 1163—Institut Imagine) Hugo Mouquet, Cliona O'Farrelly (Trinity College Dublin), Etienne Patin, Sandra Pellegrini, Antonio Rausell (INSERM UMR 1163—Institut Imagine), Frédéric Rieux-Laucat (INSERM UMR 1163—Institut Imagine), Lars Rogge, Magnus Fontes, (Institut Roche), Anavaj Sakuntabhai, Olivier Schwartz, Benno Schwikowski, Spencer Shorte, Frédéric Tangy, Antoine Toubert (Hôpital Saint-Louis), Mathilde Touvier (Université Paris 13), Marie-Noëlle Ungeheuer, Christophe Zimmer, Matthew L. Albert (HIBIO) (co-coordinators of the Milieu Intérieur Consortium), Darragh Duffy.(co-coordinators of the Milieu Intérieur Consortium), Lluis Quintana-Murci. (co-coordinators of the Milieu Intérieur Consortium).

Additional information can be found at: http://www.milieuinterieur.fr.

## STAR★Methods

### Key resources table

**Table:** 

REAGENT or RESOURCE	SOURCE	IDENTIFIER
**Bacterial and virus strains**		

Influenza A virus H1N1	Charles River	
BCG (Immucyst)	Sanofi Pasteur	
Enterotoxin SEB	Bernhard Nocht Institute	
LPS-EB (ultrapure)	Invivogen	
Poly:IC	Invivogen	

**Biological samples**		

TruCulture whole blood stimulation assay supernatant – healthy donors	Milieu Interieur consortium	
TruCulture whole blood stimulation assay supernatant – TB patients	SATVI, South Africa	

**Critical commercial assays**		

TruCulture stimulation assays	Rules Based Medicine	

**Software and algorithms**		

Source code for the analysis		https://github.com/Translational-Immunology/oxylipins_study

### Resource availability

#### Lead contact

Further information and requests for resources and reagents should be directed to and will be fulfilled by the lead contact, Cécile Gladine (cecile.gladine@inrae.fr).

#### Materials availability

This study did not generate new unique reagents.

### Experimental model and study participant details

#### Human samples

Human samples from healthy donors came from the Milieu Intérieur Cohort (age and sex distribution is given in Table S1), which was approved by the Comité de Protection des Personnes – Ouest 6 (Committee for the protection of persons) on June 13th, 2012 and by French Agence nationale de sécurité du médicament (ANSM) on June 22nd, 2012.^56^ The study is sponsored by Institut Pasteur (Pasteur ID-RCB Number: 2012-A00238-35), and was conducted as a single center interventional study without an investigational product. The original protocol was registered under ClinicalTrials.gov (study# NCT01699893). The samples and data used in this study were formally established as the Milieu Interieur biocollection (NCT03905993), with approvals by the Comité de Protection des Personnes – Sud Méditerranée and the Commission nationale de l’informatique et des libertés (CNIL) on April 11, 2018. For the tuberculosis (TB) pilot study, 25 healthy adults (age and sex distribution is given in Table S1) with asymptomatic, latent Mtb infection (LTBI), defined by a positive QFT In-Tube (QFT+) assay (Qiagen, Germany), and 25 adults without human immunodeficiency virus (HIV) with TB disease (age and sex distribution is given in Table S1), defined by a positive sputum XpertMTB/RIF test (Cepheid, USA) were identified and recruited at the South African Tuberculosis Vaccine Initiative (SATVI), Worcester, South Africa.^16^ The TB clinical study, protocols, and informed-consent forms were approved by the Human Research Ethics Committee of the University of Cape Town (ref. 234/2015).

### Method details

#### TruCulture whole blood stimulations

TruCulture whole blood stimulations were performed as previously described.^15^ Briefly, tubes were prepared in batch with the indicated stimulus, resuspended in a volume of 2 mL buffered media, and maintained at −20°C until time of use. Stimuli in this study included lipopolysaccharide (LPS, 10 ng/mL) derived from E. coli O111:B4 (Invivogen), vaccine-grade poly I:C (pIC, 20 μg/mL) (Invivogen), live Bacillus Calmette-Guerin (Immucyst, Sanofi Pasteur), live H1N1 attenuated influenza A/PR8 (IAV, 100 HAU) (Charles River), a superantigen Enterotoxin (SEB, 0.4 μg/mL) (Bernhard Nocht Institute), and a Null control. 1 mL of whole blood was distributed into each of the prewarmed TruCulture tubes, inserted into a dry block incubator, and maintained at 37°C room air for 22 h. At the end of the incubation period, tubes were opened and a valve was inserted in order to separate the sedimented cells from the supernatant and to stop the stimulation reaction. Liquid supernatants were aliquoted (for paired oxylipin and cytokine analysis) and immediately frozen at 80°C until the time of use.

#### Oxylipin analysis

Free (i.e., non esterified) oxylipins were quantified in TruCulture supernatants with LC-MS/MS as described previously.^57^^,^^58^ Sample analysis was performed in a blinded fashion with regards to the stimulation condition and samples were randomized. Briefly, 130 μL of the TruCulture supernatants were extracted by solid phase extraction (SPE) following protein precipitation with methanol. A detailed description of the SPE procedure was previously described.^57^^,^^58^^,^^59^^,^^60^ Samples from the healthy cohort and from the TB pilot study were analyzed by two different MS platforms. In the healthy cohort, 167 oxylipins were analyzed by LC-ESI(−)-MS/MS (5500 QTRAP mass-spectrometer, Sciex, Germany) (Table S2) and MS data integration was performed using MultiQuant software (version 2.1.1, Sciex) The transitions used for quantification as well as the calibration range and preparation of standards solution is reported in.^57^^,^^58^^,^^60^ Among the 167 oxylipins analyzed, 67 could be quantified (i.e., >LLOQ). Of note no so called specialized pro-resolving mediators (SPM) which formation and biological role is currently a matter of debate^61^ could not be detected in the TruCulture whole blood stimulations. Data analysis of the unprocessed dataset identified a batch effect on the data (two clusters, of 103 and 42 data points with a good balance between the stimuli), with the identified clusters matching with a change of laboratory technicians during the original clinical collection. The batch effect was corrected using the limma R package (version 3.48.3). In the TB pilot study, 136 oxyilipins were analyzed by LC-ESI(−)-MS/MS (G6460 Triple quadrupole mass-spectrometer, Agilent technologies, USA) (Table S2) and MS data integration was performed using Mass Hunter Quantitative analysis software (Agilent Technologies, USA). Among the 136 oxylipins analyzed, 21 could be quantified (i.e., >LLOQ), with an exception of 10-HDHA which was not quantifiable in the BCG stimulation of LTBI donors. No batch effect was identified. In both studies, if an oxylipin presented more than 30% of missing data (i.e., <LLOQ) in each individual group, this oxylipin was excluded from the data matrix.

#### Cytokine analysis

TruCulture supernatants were analyzed with Luminex xMAP technology as previously described.^15^

### Quantification and statistical analysis

All the data presented were log-transformed, and analysis were performed using R 4.1.0 and the tydiverse libraries. Plots were produced using the plotly and ggplot2 libraries. Principal Component Analysis (PCA), a multidimensional dataset scaling technique, was performed using the prcomp method from the stats package part of R 4.1.0. Multiple Factor Analysis (MFA), which is an extension of the PCA method, tailored to handle different blocks of data obtained from the same observations^18^ (one block constituted by 33 cytokines and one block constituted by 63 oxylipins), was used to analyze the combined dataset of oxylipins and cytokines. It was achieved on centered log-transformed data, using the MFA function from the FactoMiner library.^62^ Unsupervized classifications were performed on MFA’s molecule coordinates by agglomerative (“bottom-up”) Hierarchical Cluster Analysis (HCA), with the hclust function from the fastcluster package,^63^ using euclidean distance as metric and by applying Ward’s method for class clustering. Composition of clusters obtained with MFA-HCA by separated stimuli were compared by Multiple Correspondence Analysis (MCA) followed by HCA performed on the MCA’s molecules coordinates. MCA was performed with the MCA function of the FactoMiner Library. Dendrogram distances between clusters obtained from the MCA-HCA were then computed and used to build a graphical representation of the molecular network of oxylipins and cytokines, which was created with Cytoscape^20^ 3.9.1.

### Additional resources

The Milieu Intérieur Cohort (age and sex distribution is given in Table S1), was approved by the Comité de Protection des Personnes – Ouest 6 (Committee for the protection of persons) on June 13th, 2012 and by French Agence nationale de sécurité du médicament (ANSM) on June 22nd, 2012. The study is sponsored by Institut Pasteur (Pasteur ID-RCB Number: 2012-A00238-35). The original protocol was registered under ClinicalTrials.gov (study# NCT01699893). The samples and data used in this study were formally established as the Milieu Interieur biocollection (NCT03905993), with approvals by the Comité de Protection des Personnes – Sud Méditerranée and the Commission nationale de l’informatique et des libertés (CNIL) on April 11, 2018.

For the tuberculosis (TB) study, protocols, and informed-consent forms were approved by the Human Research Ethics Committee of the University of Cape Town (ref. 234/2015).

## Data Availability

• Data: raw datasets can be found in the supplementary data (Table S5) • Code: R code used for the analysis is available at the following address: https://github.com/Translational-Immunology/oxylipins_study • Any additional information required to reanalyze the data reported in this paper is available from the lead contact upon request.

## Supplemental information

Document S1. Figures S1–S4, Tables S1 and S4

Table S2. List of oxylipins analyzed in the study as described in the paragraph Oxylipin analysis Analyzed by LC-ESI(−)-MS/MS in the Truculture supernatants of healthy donors (MS platform #1,6500 QTRAP mass-spectrometer, Sciex) and of patients with latent or active tuberculosis (indicated with∗, MS platform #2, G6460 Triple quadrupole mass-spectrometer, Agilent technologies).

Table S3. Descriptive statistics of the raw data presented in the Method Detail part Median (lower/upper quartiles], CV and p-value (paired sample Wilcoxon test, stimulated vs. Null, FDR adjusted). Significance threshold: ∗p < 0.001.

Table S4. Raw oxylipin and cytokine datasets from the Milieu Interieur and the Tuberculosis studies
