# Supplementary material for: Integrated analysis of whole blood oxylipin and cytokine responses after bacterial, viral, and T cell stimulation reveals new immune networks
Source: iScience. 2023 Jul 18;26(8):107422. doi: 10.1016/j.isci.2023.107422 (PMC10415927; doi:10.1016/j.isci.2023.107422)
Supplement: Document S1. Figures S1–S4, Tables S1 and S4 [file mmc1.pdf]

iScience, Volume *26*

Supplemental information

Integrated analysis of whole blood oxylipin and cytokine responses after bacterial, viral, and T cell stimulation reveals new immune networks

Etienne Villain, Aurélie Chanson, Malwina Mainka, Nadja Kampschulte, Pauline Le Faouder, Justine Bertrand-Michel, Marion Brandolini-Bulon, Bruno Charbit, Munyaradzi Musvosvi, Nicole Bilek, Thomas J. Scriba, Lluis Quintana-Murci, Nils Helge Schebb, Darragh Duffy, Cécile Gladine, and the Milieu Interieur Consortium

Figure S1. Principal components composition of the global PCA (figure 1).

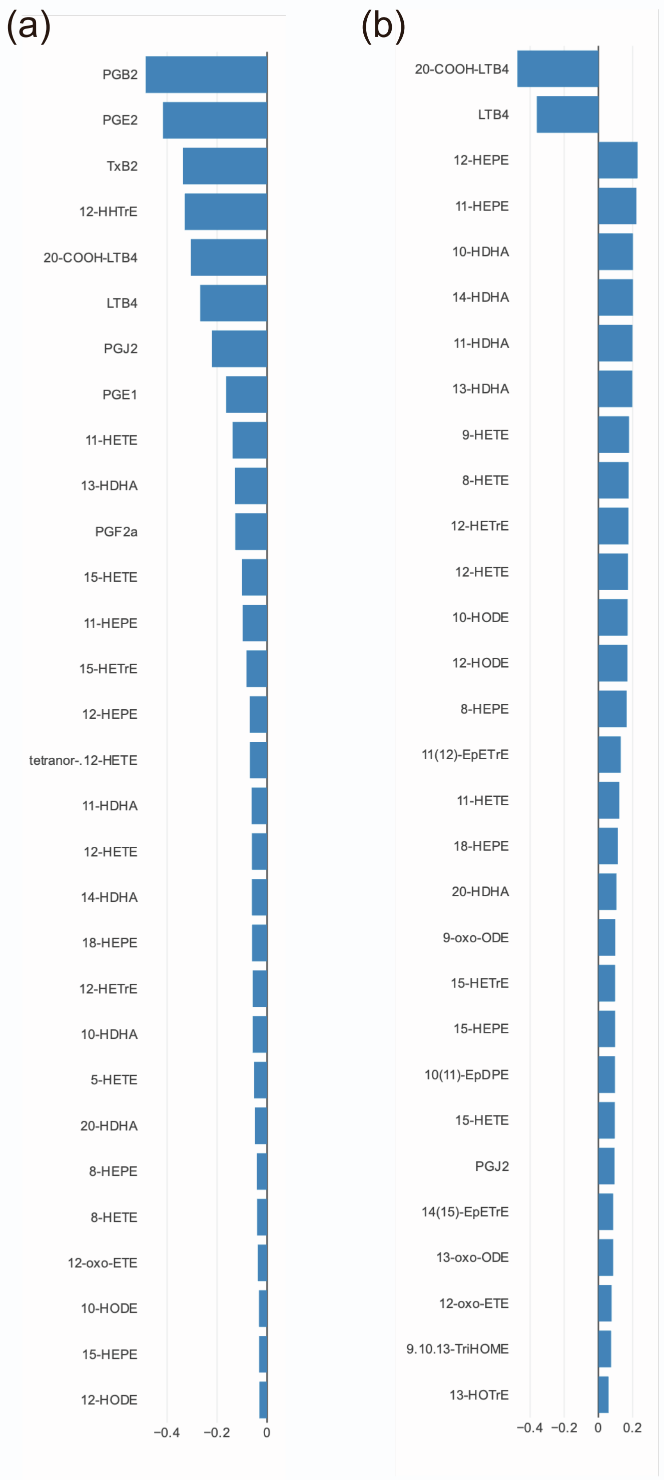

Relative contributions of the top 30 contributing oxylipins to (a) PC1 and (b) PC2 of the PCA shown in Figure 1a.

Figure S2. Dendrograms of the hierarchical agglomerative cluster analysis (expanding figure 4 with the other stimulations).

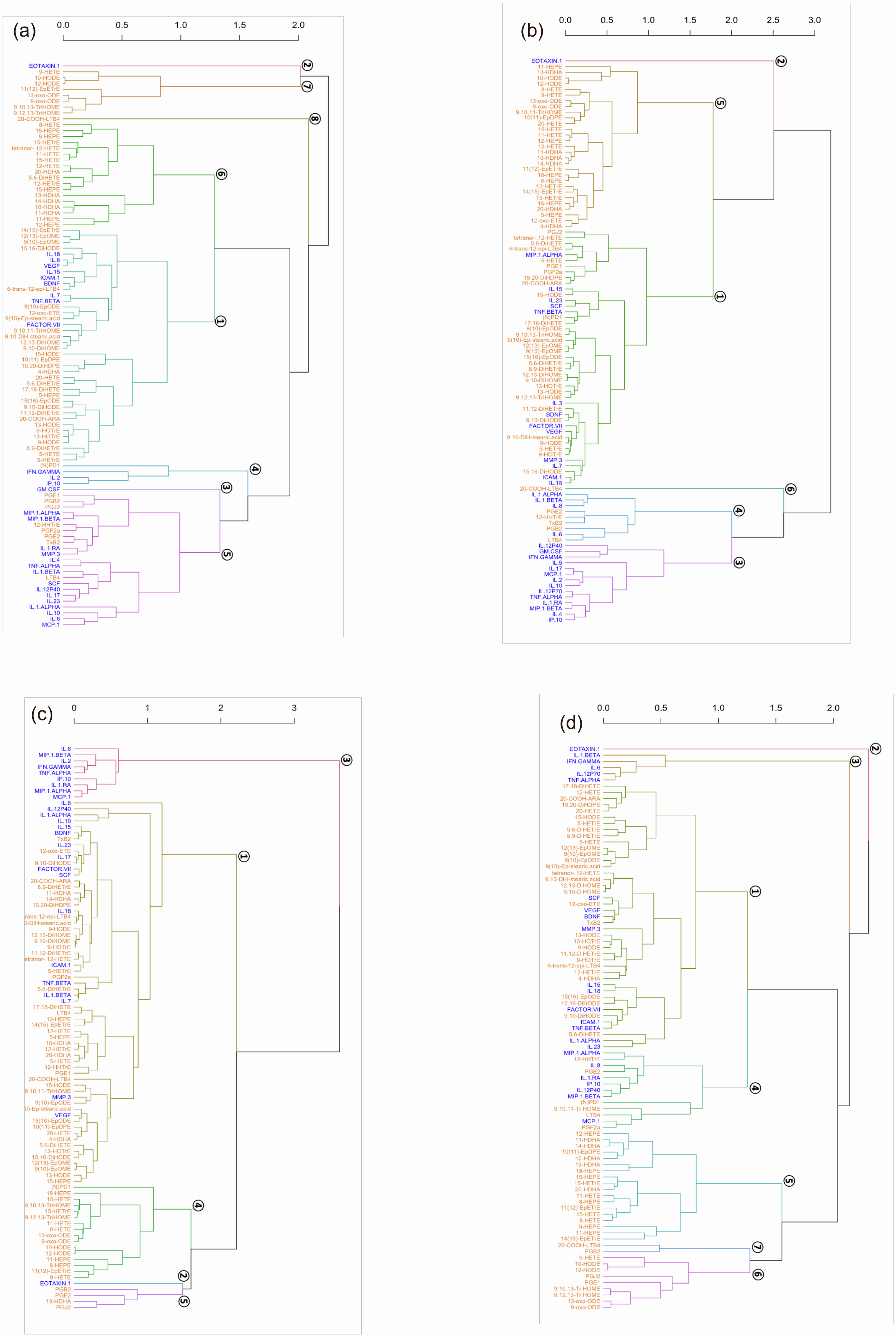

Dendrograms representing the clustering of the classes obtained in the hierarchical agglomerative cluster analysis performed on the coordinates of the variables (cytokines and oxylipins) in the first dimensions of the Multiple Factor Analysis (MFA) applied on the sub-data set corresponding to the (a) BCG, (b) SEB, (c) Influenza, and (d) Poly I:C stimuli. Depending on the stimuli, the first four [(b) & (c)] or five [(a) & (d)] dimensions of the MFA have been used, which represent 59-65% of the explained variance. Dendrograms were obtained by using the euclidean distance as metric and by applying Ward's method for class clustering. (a) Nine groups of molecules (involving 87 oxylipins and cytokines in total) were found to clusterize systematically together following the two bacterial stimuli. Numbers for the corresponding nine groups are shown in the column "# Homogeneous cluster". NA(stable) : molecules that do not react to the tested stimulus, i.e. whose concentrations are identical for all individuals.

Figure S3. Dendrograms of the hierarchical agglomerative cluster analysis for bacterial stimuli (figure 4 and S2).

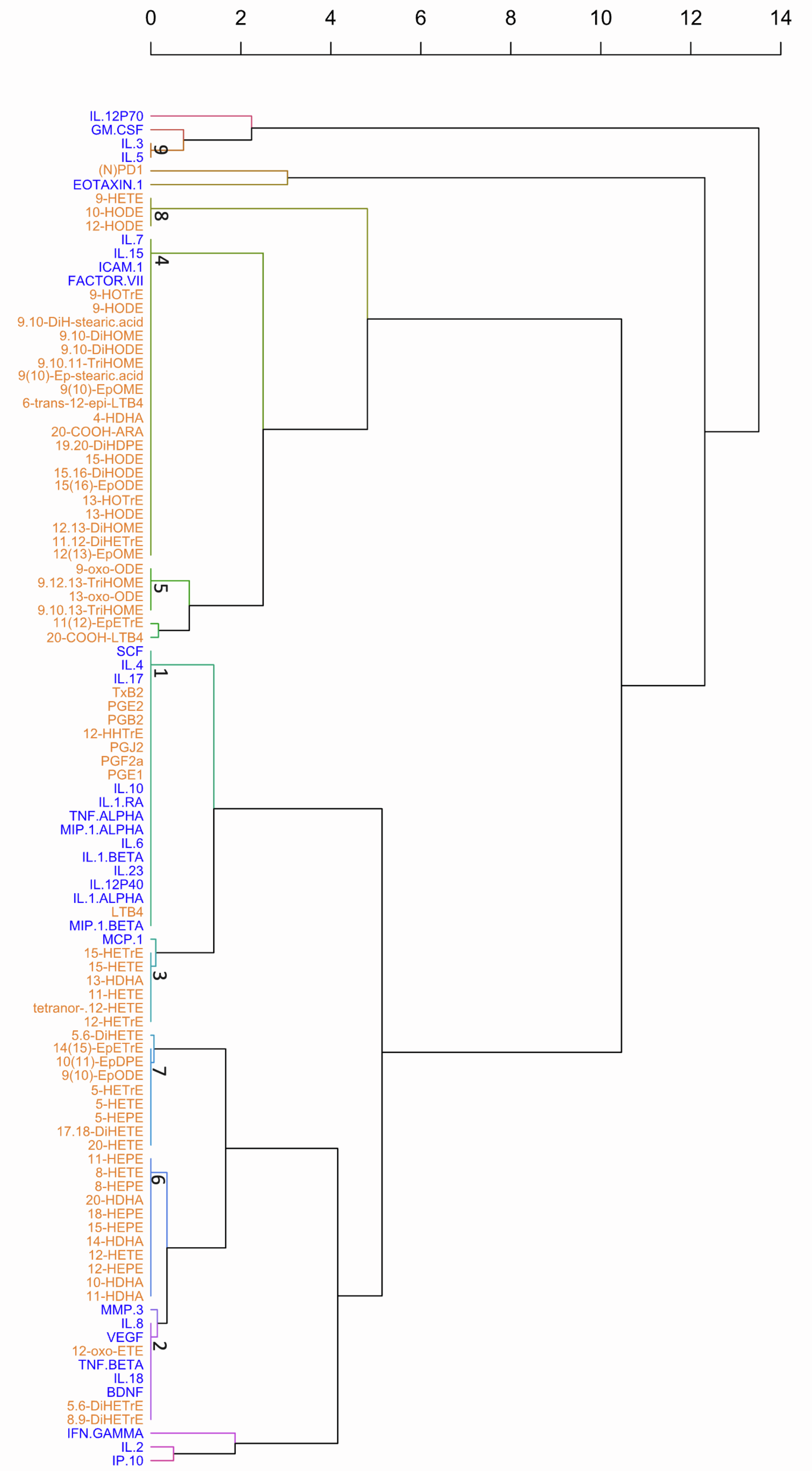

Dendrogram representing the clustering of the classes obtained in the hierarchical agglomerative cluster analysis performed on the coordinates of the variables (cytokines and oxylipins) in the first three dimensions of the Multiple Correspondence Analysis (MCA) applied on the cluster compositions generated for the bacterial stimuli (LPS and BCG), as shown in Table S4. The first three dimensions of the MCA represent 77% of the explained variance, after correction of eigenvalues according to Benzecri's formula. The dendrogram was obtained by using the euclidean distance as metric and by applying Ward's method for class clustering. Nine clusters (involving 87 oxylipins and cytokines) were identified as being similar between LPS and BCG. Corresponding numbers are indicated on the dendrogram.

Figure S4. **Proximity network analysis for the oxylipin cytokine responses (expanding figure 4 and S2).**

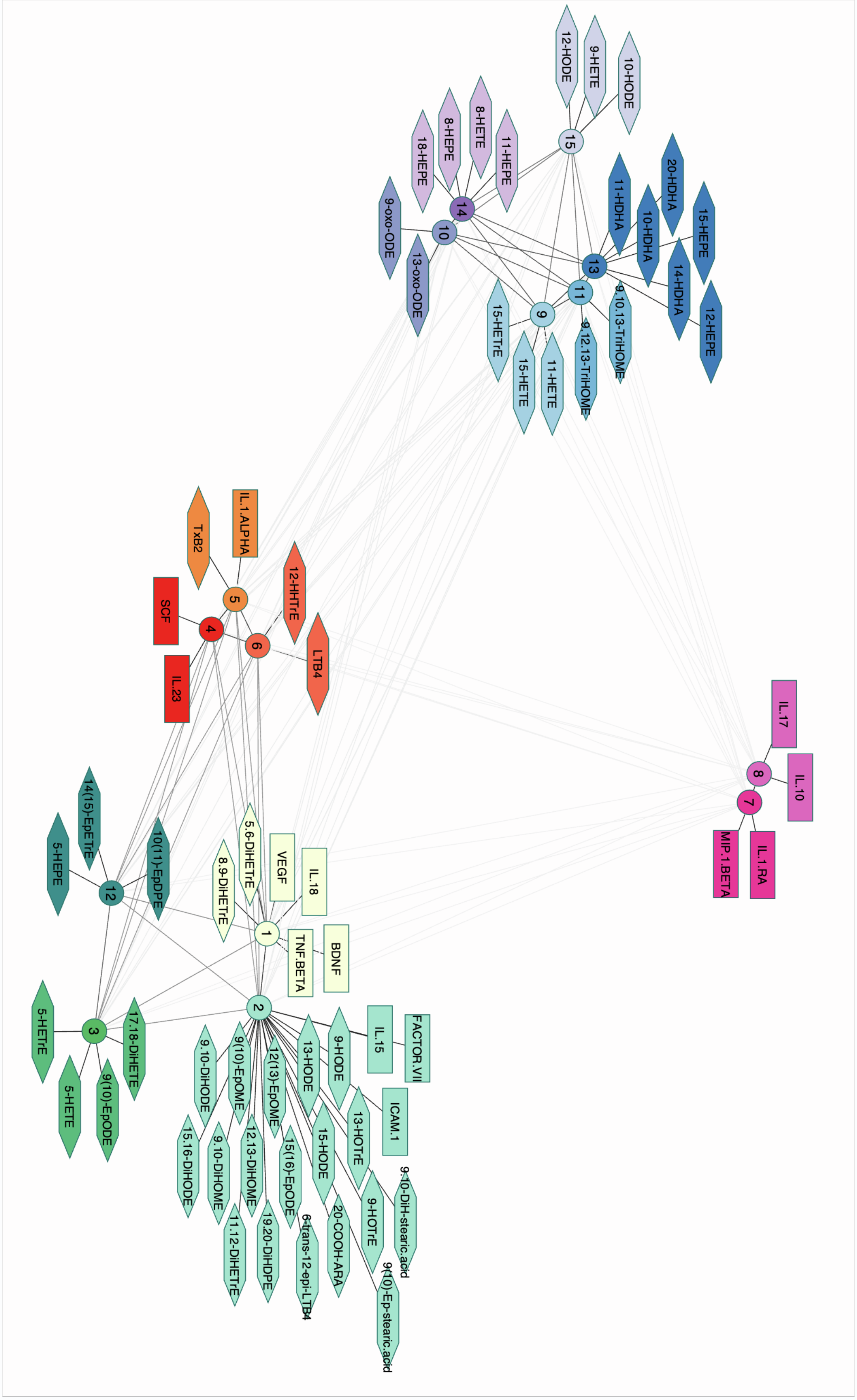

Graphical representation of the network of proximities between molecules belonging to the 15 clusters identified as similar between the LPS, BCG, SEB, Influenza and Poly-IC partitions. Distances between clusters (the darker the line, the smaller the dendrogram distance) are proportional to the inter-cluster dendrogram distances which have been calculated from the dendrogram representing the clustering of the classes obtained in the hierarchical agglomerative cluster analysis performed on the coordinates of the variables (cytokines and oxylipins) in the first three dimensions (73% explained variance, after Benzecri's correction) of the Multiple Correspondence Analysis (MCA) applied on the cluster compositions generated from the five partitions by separated stimuli, as described in Figure 5 and Figure S2. Cytokines are shown as rectangles and oxylipins as hexagons. All molecules related to a cluster are equidistant to that cluster but have been placed differently to improve visualization.

Table S1. Age and sex of included donors (related to the human samples description).

	Milieu Interieur cohort	SATVI tuberculosis patients cohort	
		LTBI controls	TB patients
N	25	25	25
Ratio men/women	12/13	8/17	8/17
Age (median, range)	34 [30-39]	33 [24-41]	33 [25-40]

Table S2. List of oxylipins analyzed in the study as described in the paragraph Oxylipin analysis. Analyzed by LC-ESI(-)-MS/MS in the Truculture supernatants of healthy donors (MS platform #1,6500 QTRAP mass-spectrometer, Sciex) and of patients with latent or active tuberculosis (indicated with*, MS platform #2, G6460 Triple quadrupole mass-spectrometer, Agilent technologies).

Table S3. Descriptive statistics of the raw data presented in the Method Detail part. Median (lower/upper quartiles], CV and p-value (paired sample Wilcoxon test, stimulated vs Null, FDR adjusted). Significance threshold: * $P<0.05$, ** $P<0.01$, *** $P<0.001$.

Table S4. **Comparison of the cluster from the hierarchical agglomerative cluster analyses (Figure 5 and S2a)**. Comparison of the composition of the clusters obtained from the hierarchical agglomerative cluster analyses performed on the coordinates of the variables (cytokines and oxylipins) in the first dimensions of the Multiple Factor Analyses (MFA) applied on the sub-data sets corresponding to the bacterial stimuli (LPS and BCG), as shown in Figure 5 and Figure S2(a). Nine groups of molecules (involving 87 oxylipins and cytokines in total) were found to clusterize systematically together following the two bacterial stimuli. Numbers for the corresponding nine groups are shown in the column "# Homogeneous cluster". NA(stable) : molecules that do not react to the tested stimulus, i.e. whose concentrations are identical for all individuals.

Table S4. **Comparison of the cluster from the hierarchical agglomerative cluster analyses (Figure 5 and S2a)**.

Molecule name	Group	LPS	BCG	# Homogeneous cluster
12-HHTrE	oxylipines	1	5	1
IL.1.ALPHA	cytokines	1	5	1
IL.1.BETA	cytokines	1	5	1
IL.1.RA	cytokines	1	5	1
IL.10	cytokines	1	5	1
IL.12P40	cytokines	1	5	1
IL.17	cytokines	1	5	1
IL.23	cytokines	1	5	1
IL.4	cytokines	1	5	1
IL.6	cytokines	1	5	1
LTB4	oxylipines	1	5	1
MIP.1.ALPHA	cytokines	1	5	1
MIP.1.BETA	cytokines	1	5	1
PGB2	oxylipines	1	5	1
PGE1	oxylipines	1	5	1
PGE2	oxylipines	1	5	1
PGF2a	oxylipines	1	5	1
PGJ2	oxylipines	1	5	1
SCF	cytokines	1	5	1
TNF.ALPHA	cytokines	1	5	1
TxB2	oxylipines	1	5	1
5.6-DiHETrE	oxylipines	1	1	2
8.9-DiHETrE	oxylipines	1	1	2
BDNF	cytokines	1	1	2
IL.18	cytokines	1	1	2
TNF.BETA	cytokines	1	1	2
12-oxo-ETE	oxylipines	1	1	2
VEGF	cytokines	1	1	2
IL.8	cytokines	1	1	2
tetranor-.12-HETE	oxylipines	1	6	3
12-HETrE	oxylipines	1	6	3
11-HETE	oxylipines	1	6	3
13-HDHA	oxylipines	1	6	3
15-HETE	oxylipines	1	6	3
15-HETrE	oxylipines	1	6	3
11.12-DiHETrE	oxylipines	3	1	4
12(13)-EpOME	oxylipines	3	1	4
12.13-DiHOME	oxylipines	3	1	4
13-HODE	oxylipines	3	1	4

13-HOTrE	oxylipines	3	1	4
15(16)-EpODE	oxylipines	3	1	4
15.16-DiHODE	oxylipines	3	1	4
15-HODE	oxylipines	3	1	4
19.20-DiHDPE	oxylipines	3	1	4
20-COOH-ARA	oxylipines	3	1	4
4-HDHA	oxylipines	3	1	4
6-trans-12-epi-LTB4	oxylipines	3	1	4
9(10)-EpOME	oxylipines	3	1	4
9(10)-Ep-stearic.acid	oxylipines	3	1	4
9.10.11-TriHOME	oxylipines	3	1	4
9.10-DiHODE	oxylipines	3	1	4
9.10-DiHOME	oxylipines	3	1	4
9.10-DiH-stearic.acid	oxylipines	3	1	4
9-HODE	oxylipines	3	1	4
9-HOTrE	oxylipines	3	1	4
FACTOR.VII	cytokines	3	1	4
ICAM.1	cytokines	3	1	4
IL.15	cytokines	3	1	4
IL.7	cytokines	3	1	4
13-oxo-ODE	oxylipines	3	7	5
9.10.13-TriHOME	oxylipines	3	7	5
9.12.13-TriHOME	oxylipines	3	7	5
9-oxo-ODE	oxylipines	3	7	5
10-HDHA	oxylipines	5	6	6
11-HDHA	oxylipines	5	6	6
12-HEPE	oxylipines	5	6	6
12-HETE	oxylipines	5	6	6
14-HDHA	oxylipines	5	6	6
15-HEPE	oxylipines	5	6	6
18-HEPE	oxylipines	5	6	6
20-HDHA	oxylipines	5	6	6
8-HEPE	oxylipines	5	6	6
8-HETE	oxylipines	5	6	6
11-HEPE	oxylipines	5	6	6
17.18-DiHETE	oxylipines	5	1	7
20-HETE	oxylipines	5	1	7
5-HEPE	oxylipines	5	1	7
5-HETE	oxylipines	5	1	7
5-HETrE	oxylipines	5	1	7
9(10)-EpODE	oxylipines	5	1	7
10(11)-EpDPE	oxylipines	5	1	7

14(15)-EpETrE	oxylipines	5	1	7
10-HODE	oxylipines	6	7	8
12-HODE	oxylipines	6	7	8
9-HETE	oxylipines	6	7	8
IL.3	cytokines	NA(stable)	NA(stable)	9
IL.5	cytokines	NA(stable)	NA(stable)	9

Comparison of the composition of the clusters obtained from the hierarchical agglomerative cluster analyses performed on the coordinates of the variables (cytokines and oxylipins) in the first dimensions of the Multiple Factor Analyses (MFA) applied on the sub-data sets corresponding to the bacterial stimuli (LPS and BCG), as shown in Figure 5 and Figure S2(a). Nine groups of molecules (involving 87 oxylipins and cytokines in total) were found to clusterize systematically together following the two bacterial stimuli. Numbers for the corresponding nine groups are shown in the column "# Homogeneous cluster". NA(stable) : molecules that do not react to the tested stimulus, i.e. whose concentrations are identical for all individuals.
